# Bayesian Inference of Evolutionary Histories under Time-Dependent Substitution Rates

**DOI:** 10.1093/molbev/msz094

**Published:** 2019-04-19

**Authors:** Jade Vincent Membrebe, Marc A Suchard, Andrew Rambaut, Guy Baele, Philippe Lemey

**Affiliations:** 1 Department of Microbiology, Immunology and Transplantation, Rega Institute, KU Leuven – University of Leuven, Leuven, Belgium; 2 Department of Biomathematics, David Geffen School of Medicine, University of California, Los Angeles, Los Angeles, CA; 3 Department of Biostatistics, Fielding School of Public Health, University of California, Los Angeles, Los Angeles, CA; 4 Department of Human Genetics, David Geffen School of Medicine, University of California, Los Angeles, Los Angeles, CA; 5 Institute of Evolutionary Biology, University of Edinburgh, Edinburgh, United Kingdom; 6 Fogarty International Center, National Institutes of Health, Bethesda, MD

**Keywords:** phylogenetics, Bayesian inference, evolutionary rate, molecular clock

## Abstract

Many factors complicate the estimation of time scales for phylogenetic histories, requiring increasingly complex evolutionary models and inference procedures. The widespread application of molecular clock dating has led to the insight that evolutionary rate estimates may vary with the time frame of measurement. This is particularly well established for rapidly evolving viruses that can accumulate sequence divergence over years or even months. However, this rapid evolution stands at odds with a relatively high degree of conservation of viruses or endogenous virus elements over much longer time scales. Building on recent insights into time-dependent evolutionary rates, we develop a formal and flexible Bayesian statistical inference approach that accommodates rate variation through time. We evaluate the novel molecular clock model on a foamy virus cospeciation history and a lentivirus evolutionary history and compare the performance to other molecular clock models. For both virus examples, we estimate a similarly strong time-dependent effect that implies rates varying over four orders of magnitude. The application of an analogous codon substitution model does not implicate long-term purifying selection as the cause of this effect. However, selection does appear to affect divergence time estimates for the less deep evolutionary history of the Ebolavirus genus. Finally, we explore the application of our approach on woolly mammoth ancient DNA data, which shows a much weaker, but still important, time-dependent rate effect that has a noticeable impact on node age estimates. Future developments aimed at incorporating more complex evolutionary processes will further add to the broad applicability of our approach.

## Introduction

Reliably estimating the time scale of phylogenetic histories remains a primary concern in the field of molecular evolution. By providing a historical context for ancestral relationships and biological patterns, divergence time estimates allow for insightful interpretations of evolutionary processes across a wide range of organisms. The ability to convert genetic divergence to time estimates dates back to seminal work by Zuckerkandl and Pauling (1965), who postulated the molecular clock hypothesis based on the observation of a roughly constant amino acid replacement rate in hemoglobin protein sequences from various species. The rate constancy hypothesis has inspired a rich development of statistical models and inference tools in order to incorporate increasingly complex evolutionary processes. Together with an explosive accumulation of genetic sequence data, this has resulted in widespread applications of molecular dating ranging from macroevolutionary processes of speciation to short-term outbreak dynamics—and even within-host evolution—of viruses.

Various approaches have been proposed to abandon the restrictive assumption of a “strict” molecular clock that enforces a single substitution rate across all phylogenetic branches. The development of “relaxed” molecular clocks includes the prior specification (Yoder and Yang 2000) or posterior identification (Drummond and Suchard 2010) of a limited number of different substitution rates across different branch partitions, various ways of modeling the change in substitution rate as an evolutionary process on a tree (autocorrelated relaxed clocks; Kishino et al. 2001; Aris-Brosou and Yang 2003), more general processes of continuous rate change across branches (uncorrelated relaxed clocks; Drummond et al. 2006), or combinations thereof (Vrancken et al. 2014).

Molecular clock models offer various stochastic descriptions of the regularity of their tick rates, but importantly, different ways also exist to calibrate the intensity of tick rates in such models. Although fossil calibrations are ubiquitously used to calibrate internal nodes in macroevolutionary trees, different sampling times can also be included as tip calibration for measurably evolving populations. The latter is routinely used in phylodynamic analyses of fast-evolving pathogens over short time scales, but almost simultaneously, the field of ancient DNA has also adopted this concept. Sophisticated molecular dating using various sources of calibration has shed light on important evolutionary questions across widely varying biological scales, including, for example, about controversies between fossils and molecular data (Dos Reis et al. 2016), host-specific viral evolutionary rates and its correlates (Streicker et al. 2012; Worobey et al. 2014), and short-sighted virus evolution (Vrancken et al. 2014).

The widespread application of molecular dating has led to the general realization that rate estimates may vary with the time frame of measurement. Specifically, by analyzing mitochondrial data sets for avian and primate species with varying fossil calibration points, Ho et al. (2005) found that rate estimates based on recent calibration points were much higher than those calibrated by older nodes. These results led the authors to suggest that empirically characterized relationships between the estimated rate and the depth of the calibration could be used as a tool for correcting divergence date estimates. The time-dependency of evolutionary rate estimates, which was also in line with the discrepancy between rate estimates directly measured from pedigrees and those inferred by phylogenetic analysis, has subsequently been revealed in several analyses of mitochondrial DNA (as compiled by Ho et al. [2015]), spanning a broad range of taxa, including insects (Papadopoulou et al. 2010; Ho and Lo 2013), penguins (Subramanian et al. 2009), fish (Genner et al. 2007; Burridge et al. 2008), and amniotes (Molak and Ho 2015). However, this time-dependent rate (TDR) phenomenon has also been criticized as an artifact of analyzing short sequences (Debruyne and Poinar 2009) and for a lack of theoretical support and empirical evidence (Emerson and Hickerson 2015), claiming that the observed rate variation resulted from artifacts and biases in rate estimation methodology. Ho et al. (2015) countered these criticisms by pointing out compelling evidence for TDR in an extended variety of taxonomic groups, including viruses (Li et al. 2007; Gibbs et al. 2010; Duchêne et al. 2014; Aiewsakun and Katzourakis 2015a, 2015b) and bacteria (Comas et al. 2013; Biek et al. 2015).

The demonstration of TDR through independent analyses of different data sets that are calibrated separately at different time depths also calls for the need to accommodate such rate variation when different time points are used as calibration in a single analysis. Arguably, the strongest case for this has been made by a study of foamy virus (FV) evolutionary rates (Aiewsakun and Katzourakis 2015b). The largely congruent viral and primate host phylogeny suggests a long-term cospeciation history between these retroviruses and their hosts. The application of host divergence estimates to the corresponding viral phylogenetic nodes revealed a large discrepancy between their short-term and long-term rates that could be modeled by a simple power-law rate decay model. An earlier study of primate lentiviruses (LVs) that employed a biogeographic calibration (Worobey et al. 2010) also revealed a large conflict with the more recent divergence times estimated when using tip-dated calibrations (Wertheim and Worobey 2009). Comprehensive meta-analysis of viral evolutionary rates demonstrated that these were examples of a more general phenomenon of time-dependent bias in rate estimates, which holds across multiple levels of viral taxonomy (Duchêne et al. 2014; Aiewsakun and Katzourakis 2016). For rapidly evolving viruses with dated tips and time to the most recent common ancestor (TMRCA) estimates that date back thousands of years, pervasive purifying selection may to a large extent explain the slower rates on deep internal branches (Wertheim and Kosakovsky Pond 2011). In this case, it was advocated that advanced codon substitution models are needed to more appropriately estimate relatively deep viral evolutionary histories.

Despite the widespread evidence for TDR, the inference of time-measured evolutionary histories with rate decay remains in its infancy. For the FV case, Aiewsakun and Katzourakis (2015b) proposed a pragmatic approach that involves estimating the genetic divergence from the present to different ancestral nodes under a strict clock and then using these estimates in a subsequent regression against time based on the internal node calibrations. Although this provided useful estimates to characterize TDR, it requires a misspecified model for estimating sequence divergence (using a strict molecular clock when estimating node heights in units of substitutions as a first step, Aiewsakun and Katzourakis [2015b]), ignoring several sources of error, and applying regression analyses to nonindependent data. Here, we develop a formal approach in a Bayesian statistical framework that draws direct inference from the data by adopting a flexible epoch modeling approach in which rates in an unobserved phylogeny are estimated as a function of time. We not only explore this approach using both nucleotide and codon substitution models on viral examples but also extend our analyses to ancient DNA from woolly mammoths.

## Results

### FV Cospeciation

**Fig. 1.**
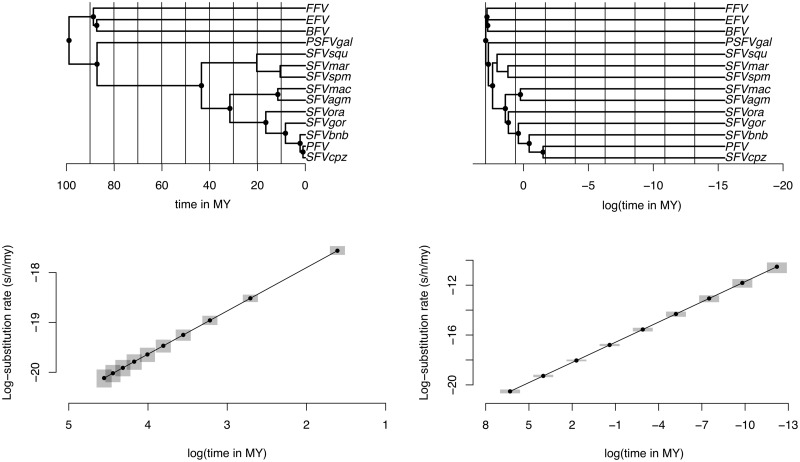
Epoch modeling in the FV phylogeny (top) and associated substitution rate estimates (bottom). Both trees are the same, but the one on the right is scaled in log time in order to better illustrate the superimposed epoch intervals (delineated by the vertical lines in both trees). The node circles in the phylogenies indicate the nodes to which a calibration prior is applied. The gray shade in the two regression plots represents the 95% highest posterior density (HPD) interval of the estimates.

Using all available host divergence time estimates as calibrations for the FV cospeciation history, we estimate a strong TDR effect under both an epoch structure with uniformly and exponentially distributed time intervals. Figure 1 depicts these epoch structures and the associated rate estimates, which illustrate a pronounced rate increase toward the present for both models in line with the regression coefficient estimates (table 1). In comparison to a previous estimate (Schweizer et al. 1999) for the short-term FV rate at 3.75 × 10^−^^4^ substitutions/site/year (s/s/yr), we estimate a lower rate close to the present in the exponential epoch model (TDR_exp_: posterior mean 2.64 [95% highest posterior density interval: 2.02–3.52] × 10^−^^5^ s/s/yr at *t_m_* = 5 years). We find an equivalent estimate at the same point in time under the uniform model to be higher (TDR_uni_: 3.81 [2.00–8.21] × 10^−^^3^ s/s/yr).

**Table 1.** Molecular Clock Model Estimates for the FV Data Set.

Clock Model	ln MLE	Parameters	Mean	95% HPD
TDR_uni_	−33,737	Intercept *β*_0_	−2.349	(−2.517; −2.157)
		Slope *β*_1_	−0.868	(−0.945; −0.800)
TDR_exp_	−33,667	Intercept *β*_0_	−3.305	(−3.389; −3.229)
		Slope *β*_1_	−0.539	(−0.570; −0.511)
Strict	−34,044	Clock rate *r*	0.012	(0.011; 0.013)
UCLD	−33,646	Mean *μ*	0.014	(0.011; 0.018)
		Dispersion *σ*	0.019	(0.014; 0.03)
RLC	−33,674	Initial rate *R*	0.0072	(0.0067; 0.0078)
		Rate changes *K*	10	(8; 12)

Note.—The uncorrelated relaxed clock model with an UCLD yields the highest log marginal likelihood estimate (ln MLE) among the clock models being compared. In order of decreasing model fit to the data, the UCLD is followed by the exponential epoch model (TDR_exp_), the RLC model, the uniform epoch model (TDR_uni_), and the strict clock model.

In contrast, the epoch models infer very slow evolutionary rates close to the root of the phylogeny (TDR_uni_: 3.08 [2.47–3.79] × 10^−^^9^ s/s/yr and TDR_exp_: 1.77 [1.05–2.94] × 10^−^^9^ s/s/yr at 99 My for both the uniform and exponential epoch model, respectively). These estimates coincide with long-term rate estimates that were obtained based on known divergence dates of the primate hosts (Switzer et al. 2005; Muniz et al. 2013).

Using (log) marginal likelihood estimates (table 1), we compare model fit for the epoch models as well as for other molecular clock models available in BEAST. The TDR_exp_ model yields a considerably better model fit compared with the TDR_uni_ model. These models essentially differ in the way the node calibration priors inform the power-law model for rates across epoch intervals. Because the calibration priors are more dispersed across the intervals in the TDR_uni_ model (fig. 1), they should be able to better inform the relationship between rate and time. The difference in model fit may therefore imply that the rate variation through time may not be as regular as expected from a power-law function over the rates in the TDR_uni_ model. The TDR_exp_ model fits significantly better than a strict clock model and somewhat better than a random local clock (RLC) model. An uncorrelated relaxed clock model with an underlying lognormal distribution (UCLD) however provides the best fit to the data. This suggests that TDR may also be modeled using branch-specific rates provided that many calibrations spread across the phylogeny can shape the rates into this pattern, which is indeed the case in this example. This is illustrated by summarizing the rate dynamics through time under the relaxed clock model in a post hoc fashion (supplementary fig. S1, Supplementary Material online). In addition, the UCLD model can also accommodate lineage-specific deviations from the strict relationship between rate and time. In fact, under simulations, we always (100%) recover the TDR_exp_ model to be a better model than the UCLD model (see Supplementary Material online), indicating that in this data set, the UCLD model is indeed accommodating the said lineage-specific deviations.

As indicated by Aiewsakun and Katzourakis (2015b), the availability of rich calibration information, as is the case for this FV example, represents an unrealistic scenario for most real-life situations. We therefore follow their performance evaluation of more realistic scenarios where only three nodes are available as calibrating nodes. We examine two of their “dispersed” and two of their “aggregated” node calibration schemes where all three calibrating nodes are of different time scales and of similar time scales, respectively (cf. Materials and Methods). In terms of model fit, the TDR_exp_ model now consistently yields the best (log) marginal likelihood estimates among all models for the dispersed calibrating schemes (table 2). The (log) marginal likelihoods are more similar across models for the aggregated calibrating schemes and the estimates do not make any meaningful distinction between the TDR_exp_ model and the UCLD model.

**Table 2.** Model Fit Estimates under Different Dispersed and Aggregated Calibrations.

Clock Model	ln MLE			
	Dispersed		Aggregated	
	I	II	I	II
TDR_uni_	−33,643	−33,667	NA^a^	−33,635
TDR_exp_	−33,629	−33,638	−33,636	−33,637
Strict	−34,691	−33,933	−33,648	−33,933
UCLD	−33,655	−33,658	−33,635	−33,636
RLC	−33,655	−33,655	−33,649	−33,636

Note.—The exponential epoch model (TDR_exp_) outperforms the other clock models for the dispersed calibrations in terms of the log marginal likelihood (ln MLE) estimates, while differences are generally less conclusive for the aggregated calibrations.

a No estimate is available for the uniform epoch model because all shallow calibrating nodes fall under a single epoch.

**Fig. 2.**
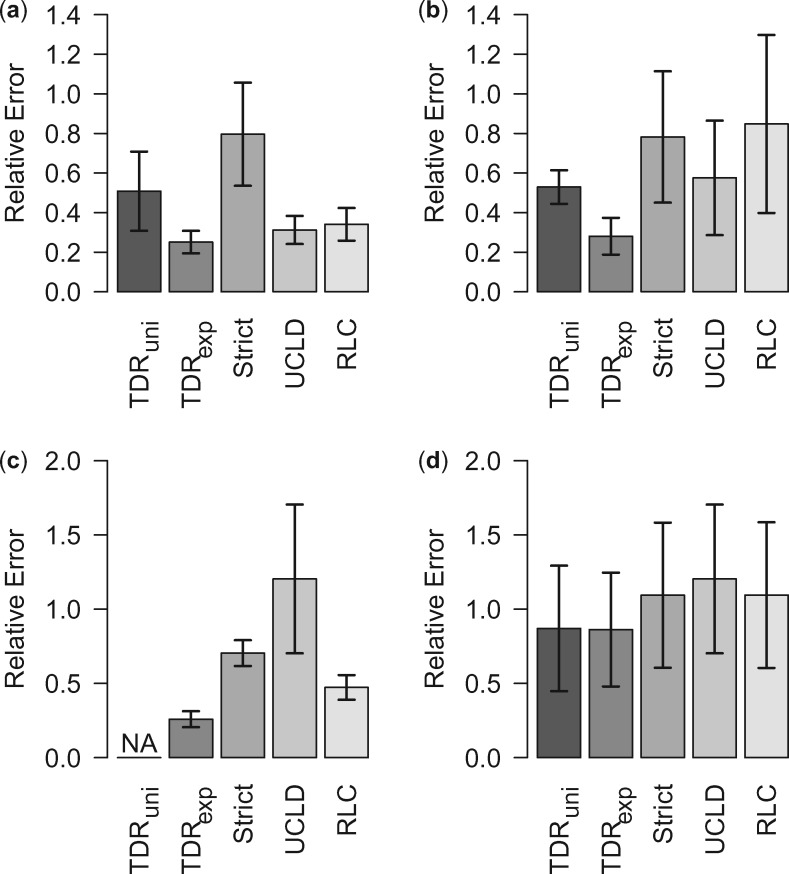
Relative errors for the different clock models under the (*a*) Dispersed I, (*b*) Dispersed II, (*c*) Aggregated I, and (*d*) Aggregated II calibration schemes. The whiskers represent the standard error of the relative rates. The exponential epoch model (TDR_exp_) is associated with the lowest mean relative error for all four calibration schemes.

In addition to model fit, we also evaluate the performance of the models in terms of node height estimation under the dispersed and aggregated calibration schemes. Assuming that the mean host divergence dates for 11 nodes represent the “true” age of the corresponding viral nodes, we summarize relative errors for age estimates across these nodes (fig. 2). For all four calibration schemes, the TDR_exp_ model is consistently associated with the lowest mean relative error.

In order to investigate whether long-term purifying selection helps to explain the TDR in the FV data set, we set up a codon substitution model with an exponential epoch structure for both the substitution rate and the nonsynonymous to synonymous substitution rate ratio (*ω*), employing all host calibrations. Although this confirms a rate decline into the past (*β*_1_ = –0.414 [–0.486, –0.343]), the TDR coefficient estimate for *ω* does not suggest any particular trend for selection through time (*β*_1_ = 5.723 × 10^−^^2^ [–0.026, 0.139]).

### LV Evolution

Using the TDR_exp_ model with two different sets of epochs (9 vs. 5, cf. Materials and Methods), we analyze a LV data set with two calibration points in the primate and human immunodeficiency virus lineage. We apply a calibration prior on the TMRCA of HIV-1 group M based on dated tip estimation (Faria et al. 2014) and another calibration prior on an older node based on biogeographic knowledge (Worobey et al. 2010). The regression coefficient estimates suggest a roughly similar TDR effect as compared with the FV evolutionary history (table 3). The model estimates a fast rate at present (TDR_exp_(9): 1.38 [1.06, 1.71] × 10^−^^3^ s/s/yr at *t *=* *5 years), consistent with dated tip estimates for the more conserved part of the HIV-1 genome (Lemey et al. 2005), and a much slower rate close to the root of the tree (TDR_exp_(9): 3.18 [2.66, 5.61] × 10^−^^7^ s/s/yr).

**Table 3.** Molecular Clock Model Estimates for the LV Data Set.

Clock Model		TMRCA (My)				
	ln MLE	Mean	95% HPD	Parameters	Mean	95% HPD
TDR_exp_(9)	−10,674	1.022	(0.6012; 1.413)	Intercept *β*_0_	−0.344	(−0.670; 0.005)
				Slope *β*_1_	−0.621	(−0.685; −0.526)
TDR_exp_(5)	−10,655	1.093	(0.623; 2.691)	Intercept *β*_0_	−0.136	(−0.643; 0.14)
				Slope *β*_1_	−0.627	(−0.660; −0.593)
Strict	−10,915	0.038	(0.031; 0.045)	Overall rate *r*	77.7	(59.9; 96.9)
UCLD	−10,856	0.032	(0.023; 0.043)	Mean *μ*	37.5	(29.5; 46.3)
				Dispersion *σ*	26.6	(21.9; 31.6)
RLC	−10,662	0.046	(0.033; 0.059)	Initial rate *R*	26.5	(19.4; 34.2)
				Rate changes *K*	4	(3; 5)

Note.—The sparse exponential epoch model (TDR_exp_(5)) yields the best log marginal likelihood estimates (ln MLE), followed by the RLC model, the denser exponential epoch model (TDR_exp_(9)), the uncorrelated relaxed clock model with an UCLD, and the strict clock model. The TDR_exp_ models yield estimates for the TMRCA that are considerably deeper than the other clock models.

By accommodating the TDR effect, the epoch models estimate a considerably deeper LV TMRCA as compared with the other clock models (table 3). This estimate is however younger than the one obtained for the same data set using the approach of Aiewsakun and Katzourakis (2016), which could be due to the use of different calibration information in addition to the different methodology. It is important to acknowledge that these TMRCA estimates only represent a lower bound because the biogeographic date used for calibration should be considered as a lower bound on the relevant viral divergence event. Both TDR_exp_ models yield a better model fit than the strict and UCLD clock models, but only the sparse TDR_exp_ model (with five epochs) provides a better model fit compared with the RLC model. In this case the RLC accommodates the two different calibrations by a rate increase on two branches in the immunodeficiency virus clade (data not shown). In line with the empirical TMRCA estimates, exploratory simulations indicate that the RLC model severely underestimates the TMRCA (see Supplementary Material online). Using a codon substitution epoch model with TDRs and *ω*’s, we recover the same TDR rate dynamic (*β*_1_ = –0.613 [–0.673, –0.546]), but no indication of long-term purifying selection as the *ω* regression coefficient even suggests somewhat higher nonsynonymous/synonymous substitution rate ratios deeper in the tree (*β*_1_ = 0.137 [0.073, 0.205]).

### Long-Term Purifying Selection in Ebola Lineages

The codon substitution analysis of the two previous data sets provided no indication that long-term purifying selection could explain the TDR effect. However, both phylogenies also extend to very deep time scales over which substitution saturation could have a profound effect and obfuscate the *ω* estimates. Therefore, we also analyze the codon substitution pattern in a viral example in which purifying selection was previously demonstrated (Wertheim and Kosakovsky Pond 2011). Specifically, we analyze the coding genes of an Ebolavirus data set representative of the Ebolavirus genus. As only dated tips and no internal calibrations are available in this case, we do not model a time-dependency for the rate but only a time-dependency for *ω*. We estimate a negative regression coefficient (table 4), demonstrating a clear decline in *ω* toward the past. This analysis results in a TMRCA estimate of 49,022 (30,586; 77,863) years which is significantly older than an estimate without time-dependency for *ω* (11,166 [7,894; 15,190] years), indicating that accounting for long-term purifying selection can have a strong impact on divergence dating. The time-dependent *ω* model also provides a better model fit to the data.

**Table 4.** Estimates for the Ebolavirus Data Set with Changing Selection Pressure (*ω*).

	Homogenous *ω*	Time-Dependent *ω*
ln MLE	−66,035	−65,767
Selection parameter(s)	*ω* = 0.028 (0.026; 0.030)	Intercept *β*_0_ = −0.559 (−0.822; −0.305)
		Slope *β*_1_ = −0.417 (−0.449; −0.385)
Clock rate (subst./site/yr)	0.0004 (0.0002; 0.0006)	0.0002 (0.0001; 0.0003)
TMRCA (years)	11,166 (7,894; 15,190)	49,022 (30,586; 77,863)

Note.—The time-dependent *ω* model resulted in a better fit compared with the homogenous *ω* model.

### Woolly Mammoths

To explore the applicability of the epoch TDR model beyond viruses, we analyze an ancient DNA data set for woolly mammoths for which a root calibration can be combined with dated tips (Chang et al. 2017). Under an exponential epoch model, we identify a TDR effect (*β*_1_ = –0.115 [–0.216, –0.007]), but less pronounced when compared with the virus examples, and with an upper credible interval that is close to, but still excludes, 0. This is associated with substitution rate estimates that vary over a much more restricted range, from 1.426 (0.386, 2.599) × 10^−^^8^ s/s/yr in the first epoch to 3.476 (2.229, 4.936) × 10^−^^9^ s/s/yr in the last epoch.

**Fig. 3.**
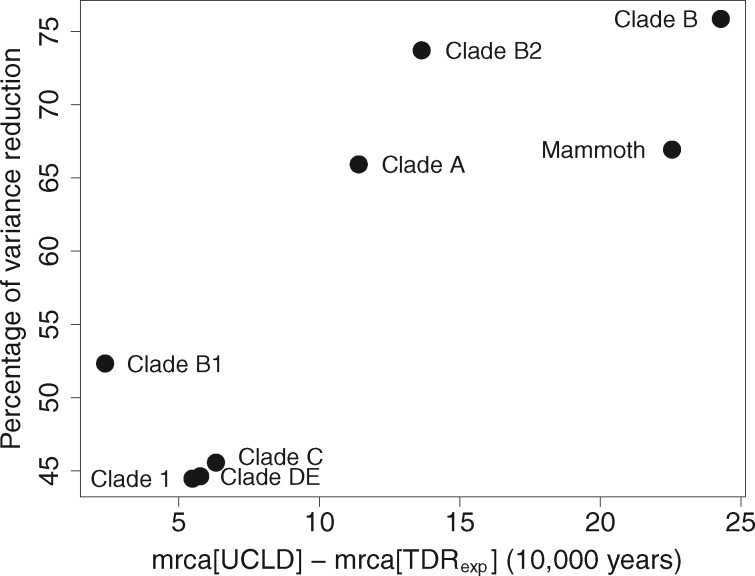
Differences in mean and variance of node age estimates between UCLD and TDR_exp_. The percentage reduction in variance of the TDR_exp_ estimates relative to the UCLD estimates is plotted against the difference in mean estimates (mrca[UCLD] − mrca[TDR_exp_]) for the node heights studied by Chang et al. (2017).

Although the TDR effect is relatively limited, it may still impact divergence time estimation. To examine this, we compare the age estimates between the TDR_exp_ and UCLD model for the different clades/haplogroups/lineages that were the focus of the original study (Chang et al. 2017). Specifically, we compare the difference in the mean estimates (mrca[UCLD] − mrca[TDR_exp_]) for the relevant nodes as well as the reduction in variance of the TDR_exp_ estimates relative to the UCLD estimates (fig. 3). Because these nodes are considerably closer to the tips than to the root, their age will be mostly determined by the recent faster rates under the TDR_exp_ model. Figure 3 confirms that this leads to more recent TDR_exp_ age estimates and that they are also associated with a considerable reduction in variance, despite the relatively limited TDR effect. The impact on the mean and variance of the age estimates is most pronounced for the deeper nodes.

## Discussion

Building on the work of Aiewsakun and Katzourakis (2015b), we developed a formal Bayesian phylogenetic approach that accommodates TDR in the estimation of divergence times. The TDR phenomenon is now widely recognized in various organisms, but it is particularly well established in rapidly evolving viruses that have a relatively long-term transmission history in animal and human populations (Duchêne et al. 2014; Aiewsakun and Katzourakis 2016). The longstanding circulation history of viruses in different hosts is becoming increasingly clear through various evolutionary studies, such as those on endogenous viral elements. Cospeciation examples, like the FVs we analyze in this study, illustrate that rapidly evolving viruses can show an unexpectedly high degree of conservation in deep histories. The recent discovery of viruses in ancient DNA samples confirms viral sequence conservation over long time scales. For HBV, an unexpected similarity to modern genomes had already been noticed for a 16th century mummy sample (Patterson Ross et al. 2018), and a relatively slow rate was subsequently estimated when focusing on genomes from samples that date back to the Bronze Age (Mühlemann et al. 2018).

Our method combines the concept of epoch modeling (Bielejec et al. 2014), which allows to change the parameterization of the sequence evolutionary process in an arbitrary discrete fashion over time, and a generalized linear model formulation over the epoch parameters. A quantifiable TDR effect is incorporated by using the log of the epoch midpoint time as the covariate for the log rates. Our implementation in the BEAST framework ensures that the approach can benefit from a rich selection of models, including various substitution models and tree priors, flexible calibration for internal and tip nodes, and state-of-the-art marginal likelihood estimation to assess model fit (Baele et al. 2016).

We apply our approach to two different viruses with deep evolutionary histories and different calibration information, but we estimate a roughly similar rate decline. In the FV cospeciation example, a large number of strong calibrating distributions could be applied across the evolutionary history. Under strong calibration constraints, a relaxed clock model with branch-specific rates drawn from a lognormal distribution can also provide a good fit to the rate decline. The strength of the epoch TDR model, in terms of model fit and/or relative error for node age estimates, is better illustrated in more realistic scenarios with fewer calibrations available.

Incorporating TDR through epoch modeling requires a specific epoch interval structure. In the analysis of the FV example, we compare a uniformly and exponentially distributed series of transition times. Although the calibrating nodes are better dispersed across the uniform structure in this example, we find better model fit for the exponential epoch model, which may indicate more temporal deviations from the power-law function over the rates in the uniform epoch model. This together with a better model fit of the uncorrelated relaxed clock under strong calibration constraints suggests that it may be useful to pursue more flexible TDR models, for example by combining a TDR effect with branch-specific random effects modeled according to the uncorrelated relaxed clock model approach.

We have specified epoch models with a relatively sparse number of epoch intervals. More dense epoch models can be specified, but it remains to be determined if they would offer any advantage in estimating divergence times. In our experience, the inclusion of more epoch intervals does not appear to have a noticeable impact on Markov chain Monte Carlo (MCMC) mixing. However, the comparison of different epoch intervals for the LV example indicates that it can affect model fit estimates, with more parameter-rich models being associated with lower marginal likelihoods. In practice, the epoch specification should have sufficient flexibility to allow the calibrating nodes to inform the TDR effect. The fact that there are only two calibrating nodes in the LV data set probably explains why a sparse epoch parameterization is preferred in this case. Finally, we adopted a power-law relationship between rate and time because it has been shown to provide a good fit to the evolutionary rate dynamics through time (Aiewsakun and Katzourakis 2015b), but other functions could also be explored in our Bayesian approach.

The time-dependency of evolutionary rates reflects our inability to adequately measure divergence over long time scales. Substitution models are able to correct for “multiple hits,” or multiple substitutions at the same sites, to some degree, but this may not suffice to recover relatively deep divergence. Therefore, extrapolating with fast recent rates, which are hardly affected by multiple hits, over deep branches that span less than expected sequence evolution will result in underestimated times of divergence. Long-term purifying selection may result in mostly neutral multiple substitutions along deep phylogenetic branches that are not adequately corrected for by nucleotide substitution models (Wertheim and Kosakovsky Pond 2011). Codon substitution models that model variation in *ω* among sites and among lineages have shown more promise for estimating deep divergence in coding genes (Wertheim and Kosakovsky Pond 2011), but their implementation in a Bayesian framework that focuses on time-measured trees has been lacking due to their computational burden. By modeling a time-dependent effect for *ω*, we demonstrate long-term purifying selection in the Ebolavirus genus, and by accounting for it, we indeed estimate a considerably older TMRCA compared with a time-homogeneous codon substitution model. This motivates further development of codon substitution models in BEAST, for which the computational burden can be alleviated by massively parallel likelihood computation through BEAGLE (Ayres et al. 2019). For the FV and LV evolutionary histories that date back much deeper into time and result in rates varying over several orders of magnitude, we believe saturation to be the main cause of TDR (Aiewsakun and Katzourakis 2015b, 2016). As this will be more pervasive for synonymous substitutions, it can explain why we do not find signal for long-term purifying selection in FV evolutionary history, and an even slightly increasing *ω* toward the past in the LV case. In such cases, our TDR model offers the additional correction that standard substitution models cannot offer, provided that calibrating information is available to inform how much correction is needed over time.

Long-term purifying selection and saturation, which are only two of the possible causes of TDR (for a review we refer to Ho et al. [2011]), may particularly impact evolutionary rate estimation for rapidly evolving viruses with small genome sizes. An obvious consequence of the fast evolutionary rate is that multiple substitutions will occur more frequently at the same sites per unit of time, and saturation will grow problematic on much shorter time scales. In addition, small genome sizes and phenotypic constraints on sequence space may allow for only limited neutral variation, enforcing viruses to evolutionarily “pace a small cage” as argued by Belshaw et al. (2008). Phenotypic constraints however do not necessarily remain constant in evolutionary history as adaptation to new hosts for example may open up new pathways in sequence space. This has recently been discussed in an opinion piece by Simmonds et al. (2019a), which has already sparked further debate on the topic (Holmes and Duchêne 2019; Simmonds et al. 2019b).

We estimate a substantially lower TDR in the woolly mammoth data set despite the fact that its evolutionary history has a deeper TMRCA (with an elephant outgroup) compared with our LV TMRCA estimate. The impact on age estimates for the woolly mammoth evolutionary history indicates that it may be important to take into account TDR for different organisms, in particular when dated tip calibration for ancient DNA sequences is combined with internal node calibrations based on paleontological records and when these node calibrations go back deep into evolutionary history.

In conclusion, we extend the array of molecular clock approaches in a Bayesian framework with a model that accounts for TDR and that alleviates particular restrictions of previous methods. Further developments are needed to accommodate other sources of evolutionary rate variation. Our approach may help to shed light on the deep evolutionary history of viruses, for which new opportunities arise through the research on endogenous viral elements and newly emerging ancient virus genomes, but it may also find applications more broadly across the field of evolutionary biology.

## Materials and Methods

### TDR Modeling

We build our TDR model of evolution starting from standard continuous-time Markov chain (CTMC) descriptions of molecular sequence change on phylogenetic trees. A CTMC is a Markovian stochastic process that models how individual ancestral characters changed along the tree to give rise to the observed sequences. The parameters that characterize a CTMC specify the rates of changes between pairs of character states ($q_{\mathrm{ij}}\geq 0$ for i≠j) through a substitution rate matrix, often denoted by *Q*. This rate matrix, together with a time variable *t*, is used to calculate a matrix of finite-time transition probabilities of character changes through matrix exponentiation:

(1) $$P(t)=e^{\mathrm{Qt}}.$$

Finite-time transition probabilities along each branch are required for computing the likelihood of the sequence data observed at the tips of the tree, which can be done in an efficient manner using the Felsenstein pruning algorithm (Felsenstein 1981).

Standard phylogenetic implementations usually assume CTMC processes to be time-homogeneous and time reversible. Time-reversible CTMCs satisfy detailed balance and return a stationary distribution that is independent of the starting state of the chain. In order to infer trees in time units (e.g., using tip or node calibrations), we first normalize the substitution model matrices (to one expected substitution per site per unit time) and then rescale branch lengths (in time units of the tree) by an estimable overall evolutionary rate parameter (or rate scalar, *r*) without loss of generality when computing transition probabilities.

In order to introduce time-nonhomogeneity as required for TDR modeling, we borrow from recent developments in phylogenetic “epoch” modeling (Bielejec et al. 2014). Specifically, this approach allows specifying an arbitrary sequence of unique substitution processes throughout evolutionary history, each characterized by a separate rate matrix, so that over a specific epoch interval, one of these processes is active across all of the lineages. Here, we apply the same concept to allow the overall evolutionary rate parameter (*r*) to vary in a piecewise constant manner according to the prespecified epoch structure. In our epoch specification, we consider the most recent sampling time (or all tip times for contemporaneous sequences) as time 0 (*T*_0_). Hence, time flows backward from the tips to the most recent common ancestor (MRCA) in the tree. Following this order, the *M* discretized time intervals in the epoch structure are created by boundaries at times $T_{0}<T_{1}<\cdots <T_{M-1}<T_{M}$, where *T_M_* = *∞*. In this epoch structure, epoch *m* starts at time $T_{m-1}$ and ends at time *T_m_*, and the boundaries *T*_1_ to $T_{M-1}$ determine a shift in evolutionary rate that simultaneously applies to all lineages in the tree at that point in time. We refer to Bielejec et al. (2014) for the details of the likelihood computation under such discontinuous processes, which essentially requires integrating out unobserved states at the time points at which the lineages are cut in the tree by the epoch boundaries.

Although estimating separate evolutionary rates induced by the epoch structure is possible under arbitrary epoch structures, it may prove challenging to inform many parameters without densely distributed calibrations across epochs or without a prior over the rates that formalizes a relationship between parameters in adjacent epoch intervals. Motivated by the work of Aiewsakun and Katzourakis (2015b), we extend the general epoch by modeling a power-law relationship between evolutionary rate and time. We achieve this by parameterizing each epoch rate (*r_m_*) as a log linear function of time:

(2) $$\text{log}\;r_{m}=\beta_{0}+\beta_{1}\;\text{log}\;t_{m},$$

where *β*_0_ represents the log rate at *T*_0_, *β*_1_ quantifies the time-dependent effect, and *t_m_* is taken to be the midpoint of epoch *m* [$t_{m}=(T_{m}-T_{m-1})/2$ for m≠M]. Because the last epoch *M* extends to *∞*, we assume in practice an alternative time for *T_M_* that extends the time series of the preceding boundaries in order to compute a finite last midpoint time.

We employ the general implementation of our model to allow for TDRs in both nucleotide and codon substitution processes. For the latter, we resort to the Goldman–Yang (GY) codon substitution model (Goldman and Yang 1994) which uses a 61 × 61 CTMC infinitesimal substitution rate matrix *Q*_GY_ = {*q_ij_*}. From codon *i* to codon *j*, the values of the off-diagonal elements *q_ij_* are determined by

(3) $$q_{\mathrm{ij}}=\{\begin{array}{cc} 0, & \text{more than one nucleotide substitution}\\ \pi_{j}, & \text{synonymous transversion}\\ \kappa \pi_{j}, & \text{synonymous transition}\\ \omega \pi_{j}, & \text{non}-\text{synonymous transversion}\\ \omega \kappa \pi_{j}, & \text{non}-\text{synonymous transition} \end{array},$$

where *π_j_* refers to the frequency of codon *j*, *κ* is the relative occurrence of transitions with respect to transversions, and *ω* represents the relative occurrence of nonsynonymous substitutions with respect to synonymous substitutions. As for nucleotide substitution models, *Q*_GY_ is scaled so that the average rate of substitution at equilibrium equals one (codon) substitution per site per unit time:

(4) $$-\sum_{i=1}^{61}\pi_{i}q_{\mathrm{ii}}=1,$$

and the overall rate scalar scales the process in the time units of the tree; *q_ii_* are fixed so that the sum of the rows of *Q*_GY_ are equal to zero. In our codon model applications, we also consider a potential time-dependent effect on *ω* by adopting our previous epoch modeling developments (Bielejec et al. 2014, 2016).

### Bayesian Inference of TDRs

Our Bayesian implementation in the BEAST 1.10 software package (Suchard et al. 2018) jointly models TDR on a random tree for which we assume a Yule speciation model (Yule 1925) or coalescent model as its generative process. When we specify prior distributions on node times for calibration purposes, we enforce these nodes to be monophyletic. For the parameters in the TDR model, we use the following prior specification:

(5) $$(\beta_{0},\beta_{1})∼\text{MVN}\left(\left[\begin{array}{c} 0\\ 0 \end{array}\right],\left[\begin{array}{cc} 1000 & 0\\ 0 & 2 \end{array}\right]\right),$$

where MVN(*M*, Σ) refers to a multivariate normal distribution with mean vector *M* and variance covariance matrix Σ. We approximate the joint posterior and its marginalizations using standard MCMC transition kernels, including random walk transition kernels on *β*_0_ and *β*_1_. MCMC analyses were run sufficiently long to ensure stationarity as diagnosed using Tracer (Rambaut et al. 2018). We capitalize on BEAGLE (Ayres et al. 2019) to improve the computational performance of our BEAST analyses of large data sets. BEAGLE supports computation on graphics cards in addition to standard computer processing units which is specifically effective when evaluating high-dimensional codon substitution models as it permits parallelization across its states (Ayres et al. 2019). We summarize posterior tree distributions in the form of maximum clade credibility trees and visualize these trees using FigTree (http://tree.bio.ed.ac.uk/software/figtree/; last accessed August 1, 2018). We compare model fit of different epoch model parameterizations to other molecular clock models, including a strict clock, an uncorrelated relaxed clock model with an UCLD (Drummond et al. 2006) and a RLC model (Drummond and Suchard 2010), using (log) marginal likelihood estimates obtained using generalized stepping-stone sampling (Baele et al. 2016).

### Data Sets and Model Specifications

We analyze TDR (and/or time-dependent selection pressure) in three different viral data sets and conclude with exploring our proposed model on ancient DNA data from woolly mammoths. We first analyze a FV data set from Aiewsakun and Katzourakis (2015b) that was used to demonstrate TDR by regressing estimates of genetic divergence against calibration times for different nodes in a fixed phylogeny. The data set consist of an alignment of 14 FV *pol* sequences (3,351 nt) from several primates, one bovine, one equine, and one feline host. Given that the viral tree topology almost perfectly matches that of the hosts, a long history of cospeciation has been assumed for these viruses. Therefore estimates for host divergence times have been used to calibrate the viral evolutionary history. Here, we make use of the ten internal node calibrations and one MRCA calibration presented in the original study (Aiewsakun and Katzourakis 2015b) (the black node circles in fig. 1). We consider a time scale in units of millions of years (My) and construct two different epoch structures that cover the expected depth of the viral phylogeny, one with uniform time intervals up to the last epoch (with boundaries 0<10<20<⋯<90<∞) and one with exponentially distributed time intervals up to the last epoch (with boundaries $0<10^{-5}<10^{-4}<\cdots <10^{2}<\infty$). For the codon model analysis, we apply the exponential epoch structure to both the evolutionary rate parameter and the *ω* parameter. We analyze the nucleotide data set under a general time-reversible substitution model (Tavaré 1986) and assume rate heterogeneity among sites as modeled using a discretized gamma distribution (Yang 1996). We use the codon substitution model described above for the codon data set as well as for those described below. In both cases, we use a Yule speciation prior on the tree.

We also explore model performance in scenarios where only 3 (instead of 11) calibrations are available, which is considered a more realistic scenario (Aiewsakun and Katzourakis 2015b). For this purpose, Aiewsakun and Katzourakis (2015b) presented three dispersed and three aggregated node calibration schemes. The dispersed schemes consist of three calibrating nodes that are relatively widely spread across the time scale of the evolutionary history, whereas the aggregated node schemes focus on three nodes within a restricted time span. Because one of each of these schemes resulted in convergence issues for particular clock models, we here report on the analyses of two dispersed (time range 2.17–87.18 and 0.96–88.7 My for Dispersed I and Dispersed II, respectively) and two aggregated node schemes (time range 0.96–8.30 and 11.50–31.56 My for Aggregated I and Aggregated II, respectively).

Next, we focus on a LV data set that is largely based on the one used in the study by Aiewsakun and Katzourakis (2016). The original alignment includes 34 integrase sequences (420 nt) from various primate (and human) immunodeficiency viruses as well as from more distantly related LVs such as feline immunodeficiency viruses, equine infectious anemia viruses, and small ruminant LVs. In order to better represent the HIV-1 group M diversity, we complemented the data set with nine additional sequences from five different HIV-1 group M subtypes. We consider two different calibrations in the LV analysis: 1) the time to the MRCA of HIV-1 group M, for which we use a normal prior distribution with a mean of 85 years and a standard deviation of 5 years (following Faria et al. [2014]) and 2) the divergence time between a simian immunodeficiency virus from a mainland drill and an simian immunodeficiency virus from a drill in Bioko (an island 32-km off the west coast of Africa) for which we specify an exponential prior with a mean of 1,000 years and an offset of 10,000 years reflecting estimates of time of isolation of the island from the mainland (Worobey et al. 2010). We consider a time scale in My and construct an epoch model with exponentially distributed time intervals up to the last epoch (with boundaries $0<10^{-5}<10^{-4}<\cdots <10^{2}<\infty$). For comparison, we also use a sparse epoch model parameterization with five instead of nine epochs (with boundaries $0<10^{-4}<10^{-2}<1<10^{2}<\infty$). For the codon model analysis, we apply the (denser) exponential epoch structure to both the evolutionary rate parameter and the *ω* parameter. To model the nucleotide substitution process, we partition the data according to codon position and specify separate Hasegawa–Kishino–Yano (Hasegawa et al. 1985) models on the three partitions and assume rate heterogeneity among sites as modeled using a discretized gamma distribution for each partition. We specify a Yule speciation prior on the tree.

As the final viral example, we analyze an Ebolavirus data set that contains 7 concatenated protein-coding sequences from 49 Ebolavirus genomes representative of the genus Ebolavirus (which includes Bundibugyo BDBV, Reston RESTV, Sudan SUDV, Tai Forest TAFV, and Zaire Ebolavirus EBOV species). This data set is available at https://github.com/evogytis/ebolaGuinea2014 and was previously used to illustrate difficulties with reliably rooting the EBOV clade that contains the lineage responsible for the 2013–2016 Ebolavirus outbreak in West Africa (Dudas and Rambaut 2014). This data set differs from the previous two because no internal node calibrations are available to estimate divergence times. Because only tip dates can be used for calibration, we cannot appropriately estimate a TDR in this case. However, we use it to compare time-dependent selection pressure between a phylogeny that extends to hundreds or thousands of years in the past and phylogenies that extend to My in the past as is the case for the previous two examples. At the same time, we explore the impact of modeling temporal variation in *ω* on divergence time estimates. We have selected Ebolavirus for this because it was previously also used as an example to demonstrate that long-term purifying selection may obscure the time scale of viral evolutionary histories (Wertheim and Kosakovsky Pond 2011). For the codon model analysis, we let *ω* vary in a time-dependent fashion over an epoch structure with exponentially distributed time intervals (in years) up to the last epoch (with boundaries $0<5\times 10^{1}<5\times 10^{2}<\cdots <5\times 10^{5}<\infty$). We compare this to a standard time-homogeneous codon substitution process. In all Ebolavirus analyses, we use a constant population size coalescent model as a tree prior.

Finally, we explore the application of the TDR model on a nonviral example data set that combines internal node calibration from the deep paleontological record with tip date calibration for ancient DNA sequences. Specifically, we analyze a data set of 143 complete mitochondrial genomes of mammoths sampled from across the northern hemisphere with an Asian elephant genome as an outgroup (Chang et al. 2017). The ages of each specimen included in the data set are based on radiocarbon dating or stratigraphic context. We follow their “root-and-tip-dating method” which combines these ages as dated tips with a calibration prior on the root. The latter was formalized as a normally distributed divergence time between the Asian elephant and mammoths of 6.7 My with a standard deviation of 0.55 My. We construct an epoch structure with exponentially distributed time intervals (in years) up to the last epoch (with boundaries 0 < 10^3^ < 10^4^ < 10^5^ < 10^6^ < 10^7^) in order to test for TDR. We model the nucleotide substitution process by applying an Hasegawa–Kishino–Yano model independently across two partitions (codon positions 1 + 2 and codon position 3), and assume rate heterogeneity among sites as modeled using a discretized gamma distribution for each partition. We specify a constant population size coalescent model as a tree prior.

## Supplementary Material

Supplementary data are available at *Molecular Biology and Evolution* online.

## Supplementary Material

msz094_Supplementary_Data
