# Supplementary material for: Bayesian Inference of Evolutionary Histories under Time-Dependent Substitution Rates
Source: Mol Biol Evol. 2019 Apr 19;36(8):1793–803. doi: 10.1093/molbev/msz094 (PMC6657730; doi:10.1093/molbev/msz094)
Supplement: msz094_Supplementary_Data [file msz094_supplementary_data.zip › TDR_SI.pdf]

Supplementary material: Bayesian inference of evolutionary histories under time-dependent substitution rates

Jade Vincent Membrede[1], Marc A. Suchard[2,3,4], Andrew Rambaut[5,6], Guy Baele[1] and Philippe Lemey[1*]

[1]*Department of Microbiology and Immunology, Rega Institute, KU Leuven – University of Leuven, Leuven, Belgium*

[2]*Department of Biomathematics, David Geffen School of Medicine, University of California, Los Angeles, CA, USA*

[3]*Department of Biostatistics, Fielding School of Public Health, University of California, Los Angeles, CA, USA*

[4]*Department of Human Genetics, David Geffen School of Medicine, University of California, Los Angeles, CA, USA*

[5]*Centre for Immunology, Infection and Evolution, University of Edinburgh, King's Buildings, Edinburgh, EH9 3FL, UK and*

[6]*Fogarty International Center, National Institutes of Health, Bethesda, MD, USA*

(Dated: March 28, 2019)

*Electronic address: philippe.lemey@kuleuven.be

Figures

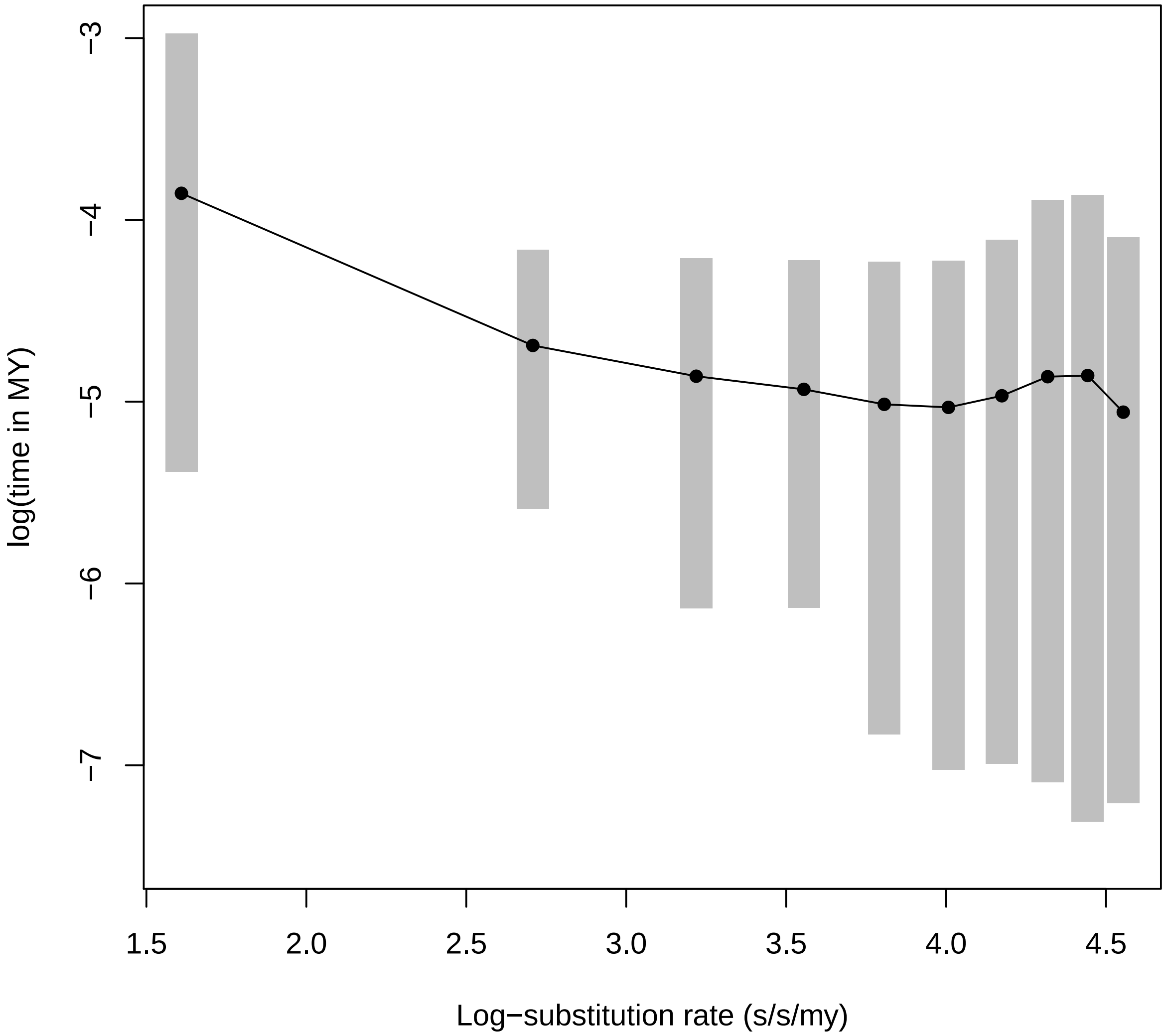

FIG. S1 Log-substitution rate estimates of the Foamy virus dataset show a declining trend over log-time under the uncorrelated relaxed clock model with an underlying lognormal distribution (UCLD). A slight increase towards the present (right-hand side) after a steady decline shows how the UCLD is able to accommodate a more complex relationship between time and substitution rate.

Simulations

We model simulations on two of the empirical data sets we analyze in our study .

Simulations according to the Foamy virus example

To compare model fit estimates for the TDR and UCLD model on data simulated under TDR, we performed simulations modelled on the Foamy virus (FV) example. We simulated 100 replicate data sets along the foamy virus (FV) tree for which 10 nodes were set to the mean heights of the calibrating distributions used in the empirical analysis (based on host divergence estimates). As a rough proxy to a continuous decline in rates, we simulate under an epoch structure with exponentially distributed time intervals (in years) up to 1 million years (with boundaries $0 < 10 < 10^2 < 10^3 < 10^4 < 10^5 < 10^6$) and uniformly distributed time intervals thereafter (with boundaries $10\text{MY} < 20\text{MY} < \cdots < 100\text{MY} < \infty$). Rate coefficients were set to the mean estimates from the empirical TDR_{exp} analysis. We used a general time-reversible (GTR) substitution model and assume that rate heterogeneity among sites follows a discretized gamma distribution. We also set these substitution parameters to the mean estimates from the TDR_{exp} analysis.

On all the replicate data sets, we fit both the TDR_{exp} clock model and the uncorrelated relaxed clock model with an underlying lognormal distribution (UCLD). By comparing marginal likelihood estimates (MLE), we found that all simulations prefer the TDR_{exp} as the best model.

Simian Immunodeficiency Virus Simulation

To explore root height estimation bias for data generated under TDR, we performed simulations modelled on the lentivirus (LV) example. We simulated 10 replicate data sets along the maximum clade credibility tree from the TDR_{exp} analysis of the empirical data set, which has a TMRCA of 1.022 MY. Similar to the simulations in the previous section, we specified an epoch structure with exponentially distributed time intervals (in years) up to 1 million years (with boundaries $0 < 10 < 10^2 < 10^3 < 10^4 < 10^5 < 10^6$) and uniformly distributed time interval thereafter (with boundaries $10\text{MY} < 20\text{MY} < \cdots < 100\text{MY} < \infty$). Rate coefficients were set to the mean estimates from the empirical TDR_{exp} analysis. We modelled the nucleotide substitution process according to an independent HKY model for two partitions (codon positions 1+2 and codon position 3), and assume rate heterogeneity among sites as following a discretized gamma

distribution for each partition. We also set these substitution parameters to the mean estimates from the TDR_{exp} analysis.

We fitted both the TDR_{exp} clock and the random local (RLC) model on these replicate data sets. For the TDR_{exp} clock, we found that the mean estimates of the TMRCA ranged from 0.879 MY to 1.012 MY. In contrast, the RLC model resulted in mean estimates of the TMRCA that ranged from 0.029 to 0.060 MY, indicating an underestimation of about two orders of magnitude.
